# Serial analysis of gene expression of lobular carcinoma *in situ *identifies down regulation of claudin 4 and overexpression of matrix metalloproteinase 9

**DOI:** 10.1186/bcr2189

**Published:** 2008-10-27

**Authors:** Dengfeng Cao, Kornelia Polyak, Marc K Halushka, Hind Nassar, Nina Kouprina, Christine Iacobuzio-Donahue, Xinyan Wu, Saraswati Sukumar, Jessica Hicks, Angelo De Marzo, Pedram Argani

**Affiliations:** 1 Departments of Pathology and Immunology, Washington University, St Louis, MO, USA; 2 Department of Oncology, Dana Farber Cancer Institute, Boston, MA, USA; 3 Departments of Pathology and Oncology, Johns Hopkins Medical Institutions, Baltimore, MD, USA

## Abstract

**Introduction:**

Although lobular carcinoma *in situ *(LCIS) has traditionally been viewed as a marker of breast cancer risk, recent clinical, pathological and genetic analyses have supported the concept that LCIS is a low risk, direct precursor of invasive lobular carcinoma. Global gene expression profiling of LCIS has not been performed.

**Methods:**

We analysed the comprehensive gene expression profile of a unique case of mass-forming LCIS using serial analysis of gene expression (SAGE). This SAGE library is publicly available online. By comparing the gene expression profile of LCIS to that of benign breast epithelium and stroma, we identified several genes up and down regulated in LCIS. Differential expression of selected genes not previously studied in LCIS was validated at the protein level by immunohistochemistry and at the RNA level by quantitative reverse transcriptase PCR (RT-PCR).

**Results:**

We identified down regulation of claudin 4 and overexpression of matrix metalloproteinase 9 in LCIS relative to normal breast epithelium and stroma. We validated these findings by immunohistochemistry in a separate series of 11 and 19 LCIS cases, respectively. Overexpression of matrix metalloproteinase 9 was further confirmed by quantitative RT-PCR analysis of the index case.

**Conclusions:**

We have created the first global gene expression profile of LCIS, and demonstrated down regulation of cell junction proteins (an expected result) and overexpression of matrix metalloproteinase 9 (an unexpected result). Additional analysis of this data made available as an online resource should facilitate further molecular characterisation of LCIS.

## Introduction

Lobular carcinoma *in situ *(LCIS) is characterised by small, discohesive epithelial cells that fill, distend and distort the terminal duct lobular units of the breast [1,2]. LCIS cells, which are cytologically identical to those of invasive lobular carcinoma, frequently contain mucin vacuoles, imparting a signet-ring cell appearance. At the immunohistochemical level, the hallmark of LCIS is loss of the expression of the E-cadherin protein, which results in the loss of cohesion of the cells. Unlike ductal carcinoma *in situ *(DCIS), a localised proven precursor to invasive breast carcinoma, LCIS tends to be multifocal and bilateral, and typically is neither calcified on mammography nor mass forming on clinical or gross pathological examination. Instead, LCIS is almost always an incidental microscopic finding identified by the pathologist. Less well-developed examples of the same process, in which there is insufficient distention and distortion of the terminal duct lobular units, are classified as atypical lobular hyperplasia (ALH).

Based on several classic studies that showed the invasive carcinomas that follow LCIS are often invasive ductal carcinomas (IDC), and that the risk of breast cancer was almost equal in each breast [3,4], LCIS has traditionally been viewed and managed as a marker of bilateral breast cancer risk. However, this concept is difficult to reconcile with several other clinical, morphological and molecular observations that suggest that LCIS is instead a cancer precursor. First, several other follow-up studies have shown that the majority (three of four) of cancers that follow ALH and LCIS are in fact ipsilateral [5-9]. Second, while many of the carcinomas that follow LCIS are IDCs, invasive lobular carcinoma is over-represented in these cases. It is possible that the frequent occurrence of IDC in patients followed for LCIS may be explained by the frequent co-existence of LCIS and DCIS in these patients, with the DCIS acting as the precursor to the IDC. In fact, when cases of pure LCIS unassociated with concurrent DCIS are studied, the invasive carcinoma that follows is almost always invasive lobular carcinoma [10]. Third, it is not uncommon for LCIS to be associated with microinvasive lobular carcinoma [11], a morphology that strongly suggests that the LCIS gives rise to the invasive lobular carcinoma.

Finally, at the genetic level, multiple studies have shown similarities between LCIS and invasive lobular carcinoma. Identical activating mutations of the E-cadherin gene have been identified in concurrent LCIS and invasive lobular carcinoma [12,13]. Array Comparative Genomic Hybridisation (CGH) [14] and mitochondrial DNA analyses have demonstrated marked similarities between matched LCIS and invasive lobular carcinoma, further supporting a clonal relationship. Moreover, LCIS demonstrates methylation of the same cancer specific genes found in DCIS, IDC and invasive lobular carcinoma [15]. Hence, many now view LCIS as a low-risk direct precursor to breast cancer that tends to be bilaterally distributed [16,17]. Under this view, the more frequent bilateral distribution of LCIS accounts for the greater proportion of contralateral cancers that follow LCIS as compared with DCIS.

Given the growing view that LCIS represents a cancer precursor, the identification of molecular alterations that suggest potential therapeutic and chemopreventive targets within this lesion is of great interest. At this point, studies have been limited by the typically small, microscopic extent of LCIS, which precludes obtaining frozen tissue with intact, high-quality DNA and RNA. At the DNA level, CGH studies have consistently demonstrated loss of the long arm of chromosome 16, where the E-cadherin gene is located [18,19]. RNA-based expression profiling analyses have not thus far been feasible.

In this study, we report the gene expression profile of a florid case of LCIS by serial analysis of gene expression (SAGE). SAGE provides an unbiased and quantitative assessment of the cellular transcriptome, and it is a reliable tool for identifying differentially expressed transcripts in cancer. In the remarkable case studied, the LCIS formed a mass lesion, such that frozen tissue could be obtained for genetic analysis. We validate several up and down regulated genes at the RNA and protein level, and identify dysregulated pathways in this unusual cancer precursor.

## Materials and methods

### Institutional Review Board approval

This study was approved by the Institutional Review Board of the Johns Hopkins Hospital. The study met the exemption criteria for human subject research because it involved the study of existing pathological specimens, and the information was recorded in such a manner that subjects could not be identified.

### SAGE library preparation

**Figure 1:**
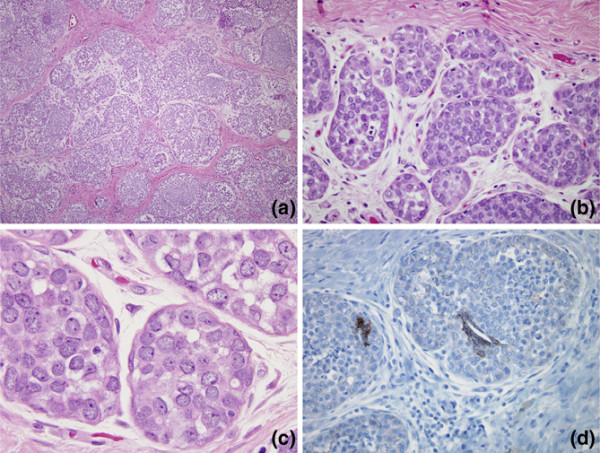
**Pathology of the index case**. (a, b) Low power view showing terminal ductal lobular units distended by small discohesive cells, typical of lobular carcinoma *in situ *(LCIS). Note the prominent perilobular oedema and fibrosis. (c) Higher power view showing discohesive nature of LCIS cells and focal intracytoplasmic vacuoles (signet-ring cells). (d) E-cadherin protein is absent in the LCIS cells by immunohistochemistry, confirming the diagnosis. Note the intact labelling in residual duct epithelial cells, providing an internal control.

The patient was a post-pubertal, pre-menopausal (further details available [20]) female who presented with a breast mass. Excision demonstrated a 5 cm, ill-defined mass that displayed the classic morphological features of LCIS. Specifically, the lobular units were filled, distended and distorted by discohesive small cells that frequently demonstrated intracytoplasmic mucin vacuoles (signet-ring cells). Immunohistochemistry for E-cadherin protein revealed an absence of labelling within the LCIS cells, with preserved membranous labelling in adjacent intact benign ductal epithelium. In some foci, the LCIS demonstrated ductal extension and focal central necrosis. Unusual features of this lesion included a relatively high mitotic rate within the lesion (average one mitosis per high power field) and focally prominent perilobular oedema and fibrosis. The latter was likely to account for the lesion being grossly evident (Figure 1). Despite extensive sampling on frozen and permanent sections, no evidence of invasion was identified. The remaining waste tissue from the optimal controlled temperature medium (OCT)-embedded cytomolds used for frozen section, along with frozen samples of ipsilateral and contralateral benign breast tissue, were the materials used for this study.

Frozen sections were prepared from several areas of the lesion and ipsilateral and contralateral normal breast tissues. On these H & E-stained frozen section slides, areas of LCIS were well delineated from the benign fibrous and fatty mammary stroma, so that they could be circled with a marking pen. No invasive carcinoma was identified on these serial frozen section slides. To enrich for LCIS cells, the OCT-embedded frozen tissue blocks from which these slides were made were macrodissected grossly, using a scalpel blade and a marked H&E-stained frozen section slide as a guide. Circled areas consisting of approximately 75% LCIS cells were manually cut out from the OCT-embedded frozen tissue blocks (Additional file 1), and RNA was extracted from these enriched samples to create the LCIS SAGE library. Additionally, normal lobules from the ipsilateral and contralateral breast were circled and macrodissected in the same fashion to enrich for epithelium for comparative quantitative reverse transcriptase PCR (RT-PCR) analysis.

SAGE provides unbiased and quantitative analysis of the cellular transcriptome. In contrast to microarrays, SAGE does not require *a priori *knowledge of the queried transcripts, and therefore allows for both quantification of gene expression and for gene discovery. SAGE libraries consist of short nucleic acid sequences or 'tags' that are concatemerised for the purposes of sequencing. SAGE tags correspond to unique transcripts or expressed sequences in the transcriptome, and the frequency of a given tag within the library corresponds to the abundance of its corresponding transcript.

Total RNA from the frozen enriched LCIS was isolated using the uMACS kit (Miltenyi Biotec, Germany) as per the manufacturer's instructions. A long (17 bp) SAGE (LSAGE) library was constructed using NlaIII as the anchoring enzyme and Mme I as the tagging enzyme, as previously described [21]. 2304 pZero-1 plasmid clones were sequenced as part of the Cancer Genome Anatomy Project's (CGAP) SAGE project [20]. The CGAP 'SAGE Tag Extraction Tool' was used to extract SAGE tags and linker sequences used in library construction. Human SAGE libraries were generated at an approximate resolution of 50,000 SAGE tags per library or more, and as a 'first-pass' filtration, tags occurring only once or with multiple annotations to the genome were removed from further analysis. Prior to analysis, all SAGE libraries were normalised to 200,000 tags.

### LCIS cases used for validation of SAGE data

Immunohistochemical labelling was performed on formalin-fixed, paraffin-embedded tissue blocks of 18 well-characterised, typical cases of LCIS, all of which were reviewed by a dedicated breast pathologist (PA), along with the index case (19 total cases). The LCIS cases featured monomorphic, discohesive cells with focal cytoplasmic vacuolisation that filled, distended and distorted lobular units. Aside from the index case, none of these typical cases demonstrated necrosis, pleomorphism or increased mitotic activity. Seven of these LCIS cases were associated with an invasive lobular carcinoma, while the other 12 were unassociated with invasive carcinoma. The four cases of this group that were analysed for E-cadherin expression by immunohistochemistry were, as expected, negative. The other cases were not analysed because their morphology was so characteristic.

### Immunohistochemistry methods

Briefly, 4 μm sections were deparaffinised in xylene for 30 minutes and rehydrated using graded ethanol concentrations. Immunohistochemical labelling was performed using the avidin-biotin peroxidase complex technique and 3',3'-diaminobenzidine as chromagen. The antibodies used, vendors, clones, retrieval methods and dilutions were as follows: matrix metalloproteinase 9 (MMP9) (Vector Laboratories, Burlingame, CA, USA, VP-M6444, clone 2C3, microwave retrieval in citrate buffer, 1:40 dilution), matrix metalloproteinase 2 (MMP2) (Labvision, Fremont, CA, USA Catalog #RB-9233-P0; microwave retrieval in citrate buffer, 1:50 dilution) and claudin 4 (Zymed Laboratories, San Francisco, USA, Clone 3E2C1, steam in citrate buffer, 1:100 dilution).

### Quantitative RT-PCR

Both normal and tumour RNA were extracted using the Trizol method, and all cDNAs were prepared with 1 μg of RNA in SuperScript II (Invitrogen, Carlsbad, CA) reactions according to the instructions of the manufacturer. Real-time SYBR green RT-PCR (Invitrogen, Carlsbad, CA) was performed on an Applied Biosystems PRISM 7900. Amplifications of MMP2, MMP9 and internal control gene *GAPDH *were performed with the following primer pairs: MMP2, forward 5'-AGATTCCAGAGAGTGGCTCCTC-3' and reverse 5'-GTAAGAAAGGTTCTAAGGCAGCC-3'; MMP9, forward 5'-CCAGCTGTATTTGTTCAAGGATG-3' and reverse 5'-CTTTCTCTCGGTACTGGAAGACG-3'; and GAPDH, forward 5'-GAAGATGGTGATGGGATTTC-3' and reverse 5'-GAAGGTGAAGGTCGGAGTC-3'.

## Results

### Identification of differentially expressed SAGE tags in LCIS

In the LCIS LSAGE library, 67,834 SAGE tags were iterated. The data is available online [20]. To determine which genes were expressed differentially between LCIS and normal breast epithelium, we used the online SAGE Digital Gene Expression Displayer (DGED) program to compare the LCIS LSAGE library with pooled LSAGE libraries prepared from normal duct epithelial cells (LSAGE-Breast-normal_epithelium_CD44+_AP_N1 (56,008 tags), LSAGE_Breast_normal_epithelium_CD24+_AP_N2 (41,430 tags), LSAGE_Breast_normal_epithelium_CD24+_AP_N1 (41,551 tags)), myoepithelial cells (LSAGE_Breast_normal_myoepithelium_AP_IDC7(69,006 tags)) and stroma (LSAGE_Breast_normal_stroma_B_IDC8 (50,485 tags)). Our rationale was that the enriched LCIS sample we used for SAGE still contained some contaminating normal breast stroma and myoepithelium, so inclusion of stromal and myoepithelial SAGE libraries in the comparison would negate their contribution and allow meaningful distinctions between LCIS cells and normal breast epithelial cells. Using this DGED analysis, 223 tags that were differentially expressed by two-fold at a p value of 0.05 were identified. Of these tags, 110 were overexpressed by two-fold, with 123 underexpressed by two-fold. We used the online Digital Northern function to demonstrate the relative expression of genes of interest from this list in these and other online LSAGE libraries derived from different breast lesions. We selected genes to validate based on biological relevance and availability of well-characterised commercial antibodies for immunohistochemistry.

### Known genes found to be differentially expressed in the LCIS LSAGE library

Initial analysis of the LCIS LSAGE library results revealed concordance with the published literature on LCIS. First, E-cadherin was noted to be down regulated compared with the three LSAGE libraries derived from normal mammary epithelium, concordant with the known inactivation of E-cadherin in almost all LCIS and the absence of E-cadherin expression in the sample used to generate this LSAGE library (Additional file 2). Additionally, alpha-catenin and delta-catenin expression was not detected in the LCIS LSAGE library, while they were abundantly expressed in the normal duct epithelial cell LSAGE libraries. These results are consistent with multiple published studies demonstrating that LCIS is associated with inactivation and disruption of the entire E-cadherin complex, including alpha-catenin, beta-catenin and p120-catenin [22-25]. Finally, cyclin D1 was noted to be overexpressed in the LCIS LSAGE library compared with normal luminal epithelium. Although initial studies had suggested the cyclin D1 was overexpressed in invasive lobular carcinoma but not LCIS [26], more recent studies have shown up regulation of cyclin D1 in LCIS [27]. We also found up regulation of cyclin D1 in four cases of LCIS relative to normal duct epithelium by immunohistochemistry (data not shown).

### Identification and validation of novel differentially expressed genes in the LCIS LSAGE library

#### Claudin 4

**Figure 2:**
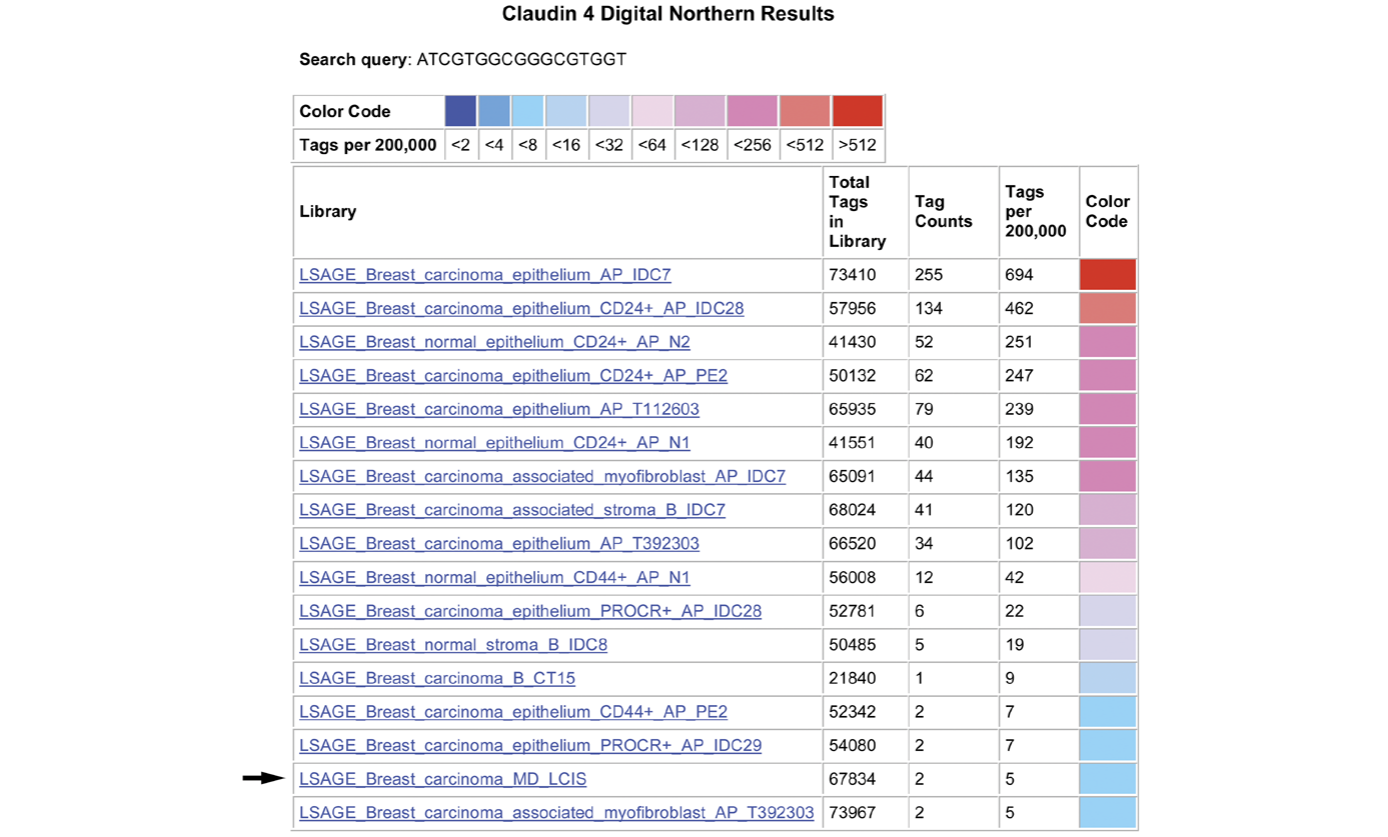
**Digital Northern blot of claudin 4 expression in breast long serial analysis of gene expression (SAGE) libraries**. Note that the lobular carcinoma *in situ *(LCIS) library shows lower expression of claudin 4 than all of the other breast epithelial and stromal libraries, with the exception of a single myofibroblast library.

**Figure 3:**
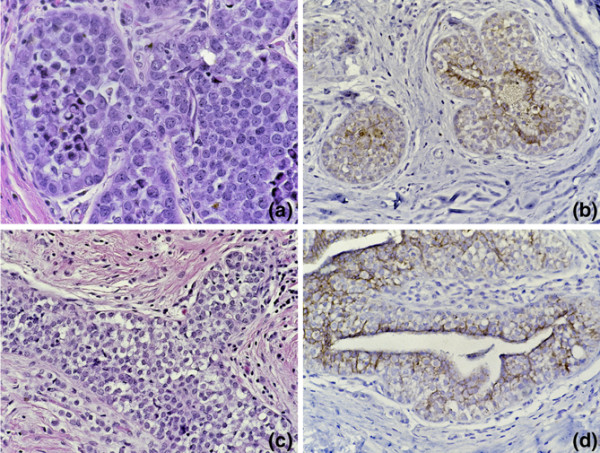
**Claudin 4 immunohistochemistry (IHC) in separate lobular carcinoma *in situ *(LCIS) cases**. (a) H & E section of LCIS case 1. (b) Claudin 4 IHC of LCIS case 1. Note the intact labelling of undermined residual strips of luminal epithelium and diminished expression in LCIS cells. (c) H & E section of LCIS case 2. (d) Claudin 4 IHC of LCIS case 2. Note the intact labelling of residual luminal epithelium and diminished expression in LCIS cells which undermine the luminal epithelium.

Claudins are tight junction proteins that are aberrantly expressed in several human cancers [28-30]. Claudin 4 was noted to be down regulated in the LCIS SAGE library compared with the five pooled normal libraries using the DGED program, and underexpressed compared with all other libraries derived from benign and malignant breast epithelium using the Digital Northern function (Figure 2). Therefore, we examined a series of well-characterised cases of LCIS for claudin 4 expression by immunohistochemistry. Claudin 4 was noted to be underexpressed compared with the normal mammary epithelium, which showed membranous labelling in nine of 11 cases of LCIS (Figure 3), with expression at similar levels in two of 11 cases. Four of these cases were associated with concurrent invasive lobular carcinoma. In two of these four invasive lobular carcinomas, claudin 4 was also underexpressed, whereas in the other two expression in the invasive lobular carcinoma was at the same level as that seen in normal mammary epithelium. Of note, E-cadherin protein expression was negative in both cases of LCIS in which claudin 4 expression was not down regulated.

**Figure 4:**
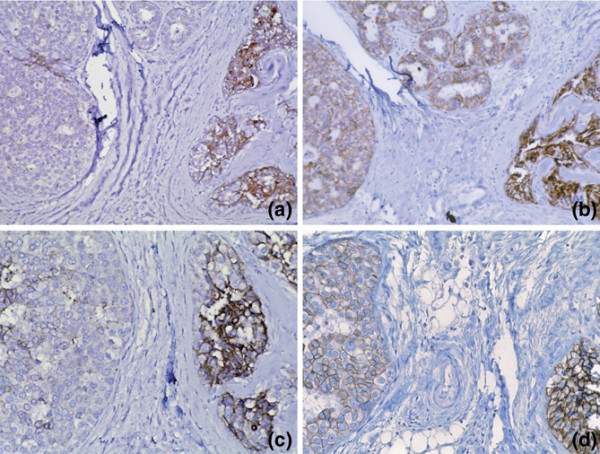
**Claudin 4 immunohistochemistry in a ductal carcinoma *in situ *(DCIS) case**. (a, c) Claudin 4 is intact in the duct of DCIS at the right, but diminished in the duct of DCIS to the left. (b, d) Expression of *E-cadherin *parallels that of claudin 4, greater in the duct to the right and diminished in the duct to the left.

For comparison, we also labelled eight cases of DCIS for claudin 4. Consistent with the prior literature [31], up regulation of claudin 4 was identified in four of eight cases, whereas in two of eight cases claudin 4 expression was at similar levels in the DCIS and normal ductal epithelium. In the other two cases, expression of claudin 4 was variable within different ducts harboring DCIS. We were able to obtain serial sections harboring these same DCIS-bearing ducts from one of these cases, and demonstrated variable E-cadherin expression in the DCIS which mirrored that of claudin 4 (Figure 4). Areas underexpressing E-cadherin also underexpressed claudin 4. Hence, in almost all LCIS and at least some cases of DCIS, the expression of claudin 4 parallels that of E-cadherin.

#### Matrix metalloproteinases

**Figure 5:**
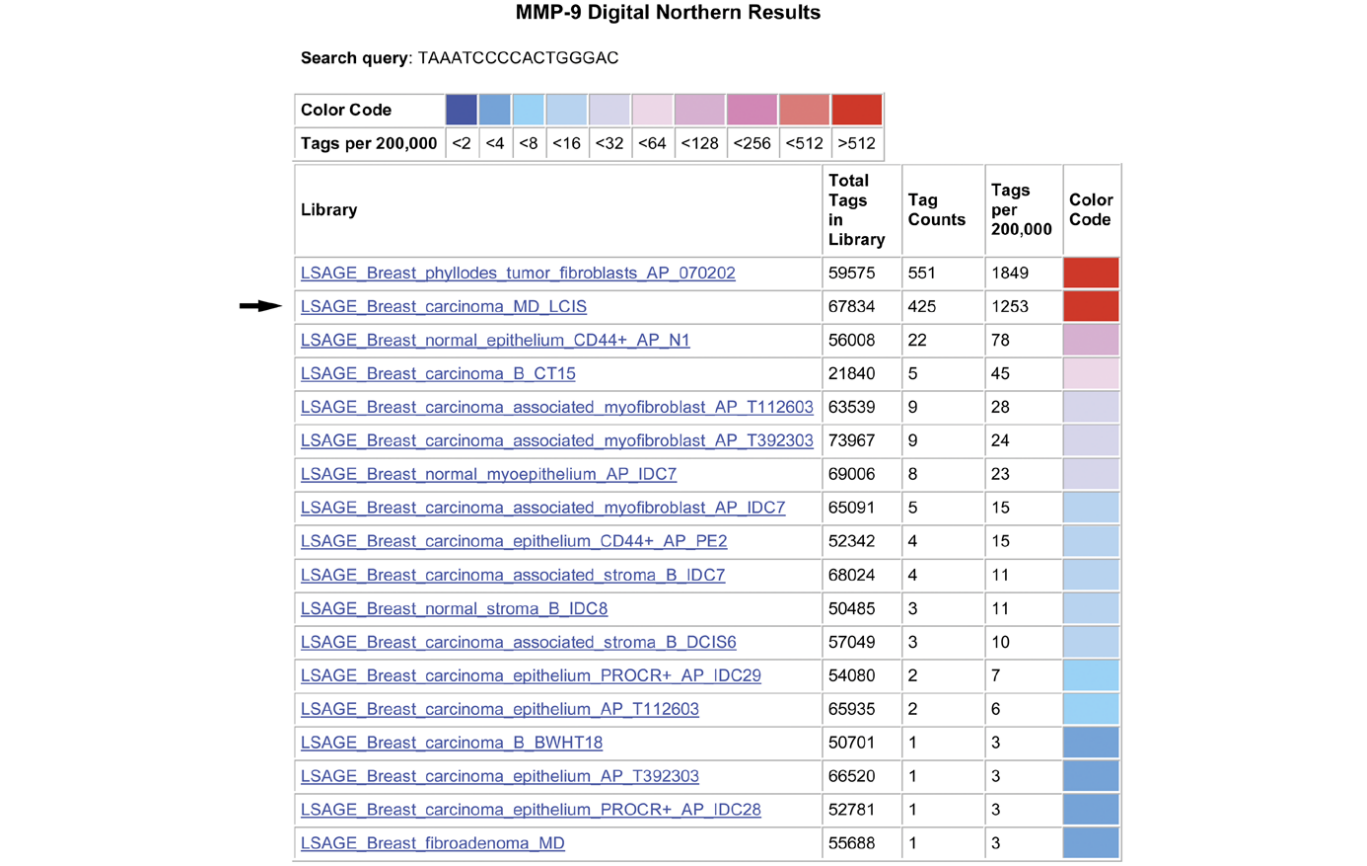
**Digital Northern blot of matrix metalloproteinase (MMP) 9 expression in breast long serial analysis of gene expression (SAGE) libraries**. Note that the lobular carcinoma *in situ *(LCIS) library shows higher MMP9 expression than all of the other breast epithelial and stromal libraries, with the exception of a single phyllodes tumour-derived library.

MMPs are postulated to be important in the degradation of basement membrane and extracellular matrix during cancer cell invasion. In particular, MMP9 and MMP2 are involved in the degradation of type 4 collagen, which comprises basement membranes, a process thought to be important in the development of invasive carcinoma and metastasis [32,33]. Using the DGED program, MMP9 was noted to be overexpressed in the LCIS LSAGE library compared with the pooled normal libraries, and overexpressed compared with all SAGE libraries derived from benign and malignant ductal epithelium using the Digital Northern function (Figure 5).

**Figure 6:**
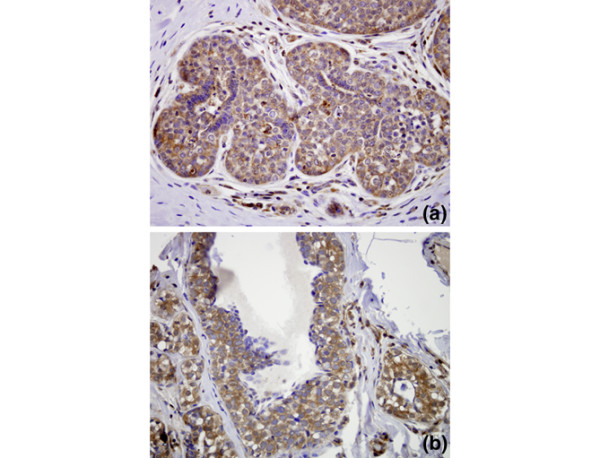
**Matrix metalloproteinase (MMP) 9 immunohistochemistry (IHC) on separate lobular carcinoma *in situ *(LCIS) cases a and b**. LCIS cells show greater immunoreactivity than strips of undermined normal duct epithelium, which are essentially negative in each case.

Therefore, we performed immunohistochemistry for MMP9 on a series of 19 cases of LCIS with adjacent normal breast epithelium. In normal lobules, MMP9 labelling was noted to be weak in the normal myoepithelium; the normal luminal epithelium was generally negative, although weak labelling was occasionally seen. Consistent with previous observations [33], strong MMP9 expression was consistently noted in macrophages comprising biopsy cavities, which were seen in several sections, and moderate labelling of the smooth muscle of medium caliber blood vessels was also appreciated. We demonstrated up regulation of MMP9 relative to normal duct luminal epithelium in 15 of 19 cases (Figure 6), with the remaining four cases of LCIS showing expression similar to that of normal luminal epithelium.

In contrast, there was a less impressive suggestion of MMP2 overexpression in LCIS relative to normal luminal epithelium using the DGED function; however, stromal and myoepithelial libraries expressed more MMP2 than the LCIS library did, raising the possibility that the small difference was artifactual and due to stromal contamination (Additional file 3). Therefore, we performed immunohistochemistry on a series of nine cases of LCIS, and found that MMP2 was overexpressed in only one case, whereas it was expressed at equivalent levels to normal epithelium in the other eight (data not shown). Hence, the immunohistochemical results for MMP9 and MMP2 correlated well with the SAGE results.

**Figure 7:**
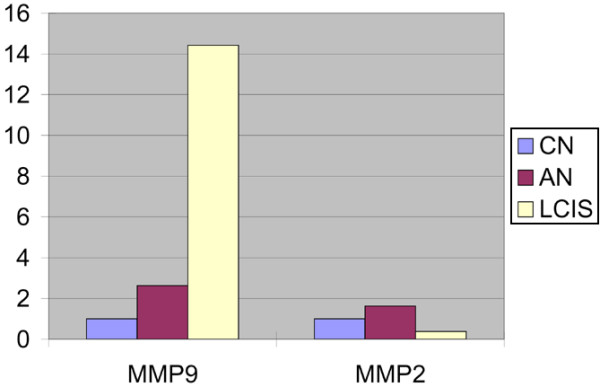
**Quantitative reverse transcriptase (RT) PCR for matrix metalloproteinase (MMP) 2 and MMP9 expression in the index case**. RNA was extracted from macrodissected lobular carcinoma *in situ *(LCIS), adjacent normal (AN) and contralateral normal (CN) lobules. Quantitative RT-PCR for MMP2, MMP9 and the housekeeping gene (GAPDH) was performed. Note the increased expression of MMP9 but not MMP2 in the LCIS versus the normal duct epithelial samples.

To further validate the overexpression of MMP9 in LCIS, we performed quantitative RT-PCR for MMP2 and MMP9 on macrodissected frozen LCIS, ipsilateral normal lobules and contralateral normal lobules from the index case. This analysis demonstrated that only MMP9, and not MMP2, was overexpressed in LCIS, again concordant with SAGE and immunohistochemical results (Figure 7).

## Discussion

SAGE is an unbiased and quantitative method of identifying differentially expressed transcripts in human cancer, enabling discovery of cancer biomarkers, therapeutic and chemopreventive targets. For example, in 2001, SAGE was used to first demonstrate that mesothelin [34] and prostate stem cell antigen (PSCA) [35] were overexpressed in pancreatic adenocarcinoma. Mesothelin and PSCA have subsequently been utilised as adjuncts in diagnosis, as candidate serum biomarkers and as targets of pancreatic cancer immunotherapy. We report herein the first SAGE analysis of LCIS. Such studies have previously not been possible because of the microscopic nature of LCIS.

The unique nature of the index case of this study, specifically the fact that it formed a mass, allowed the lesion to be identified grossly such that a small amount could be frozen for gene expression studies. Although our study is somewhat limited by the fact that the analysis is only of a single LCIS case with some unusual features (mass-forming lesion, central necrosis), the LCIS LSAGE library demonstrated differential expression of several genes (E-cadherin, cyclin D1), which was concordant with the published literature on these genes in LCIS. Moreover, we validated the results immunohistochemically for novel genes that are both up regulated (MMP9) and down regulated (claudin 4) relative to normal ductal epithelium on a larger series of more typical LCIS cases. Therefore, we believe our results are more globally applicable to LCIS and that alterations identified in other pathways not specifically validated in this study are nonetheless very likely to be significant.

One key theme of our SAGE analysis was the observed down regulation of cellular junctional proteins in LCIS. Such down regulation should have been expected. Epithelial cell junctions are comprised of both tight and adherens type junctions. Inactivation of the E-cadherin protein, a key component of the adherens junction that interacts with the actin cytoskeleton via binding of alpha, beta and delta catenins, is a hallmark of LCIS. Previous studies have demonstrated inactivation of the entire E-cadherin complex in LCIS, including loss of expression of alpha-catenin and membrane to cytoplasmic translocation of p120 catenin [22-25]. Therefore, the down regulation of E-cadherins that we observed in the index case and the four cases in the validation set is consistent with the published literature, and supports the concept that the adherens junction complex is inactivated in LCIS.

The loss of the tight junction protein claudin 4, validated by immunohistochemistry in this study, has not been previously reported in LCIS. However, these results are concordant with current knowledge of claudin biology and the known expression pattern of claudin 4 in related carcinomas. First, at the cellular level, tight junction formation is thought to depend on intact adherens-based intercellular contacts [36]. Therefore, because of the absence of E-cadherin and therefore the absence of a functional adherens junction in LCIS, the absence of tight junctions (and therefore claudin 4) should be expected. The striking parallel expression of E-cadherin and claudin 4 on serial sections of the remarkable case of DCIS analysed in this study (Figure 4) further supports the notion that the expression of E-cadherin and claudin 4 proteins is tightly linked.

Second, although no previous study has examined LCIS, several studies have examined expression of claudin 4 in invasive lobular carcinoma. Two studies reported intact claudin 4 expression in invasive lobular carcinoma [37,38]; however, these studies did not compare expression with the normal duct epithelium, and used as little as 10% labelling as a criterion for positivity. The one study that compared labelling to that of the normal duct epithelium reported down regulation of claudin 4 in two of two cases of invasive lobular carcinoma [39]. Moreoever, it has been reported that diffuse gastric-type adenocarcinoma, which cytologically and immunohistochemically resembles invasive lobular carcinoma in that it is composed of discohesive signet-ring cells that lack E-cadherin protein expression, demonstrates down regulation of claudin 4 protein [40,41]. Hence our results with LCIS are consistent with those seen in related invasive carcinomas.

Importantly, these results have therapeutic implications. Our group has shown that claudin 4 is up regulated in IDC, and is a proposed target for therapy and chemoprevention [31]. Our results show that such an approach may not be applicable to LCIS or to invasive lobular carcinoma, because these lesions typically underexpress claudin 4 relative to normal breast epithelia.

In contrast, we were surprised by the overexpression of MMPs in LCIS, and therefore we thought that it was important to validate and verify this result using multiple methods. Indeed, we show herein that MMP9 is over expressed at the RNA and protein level in LCIS by quantitative RT-PCR and immunohistochemistry, respectively. No previous study has systematically evaluated MMP9 expression in LCIS. However, one study did find overexpression in invasive lobular carcinoma relative to IDC, and noted that a single case of concurrent LCIS also showed MMP9 expression [42].

Moreover, recent data in ovarian and pulmonary carcinomas has suggested a causal inverse relationship between E-cadherin and MMP9 expression, which would fit our finding of high MMP9 expression in LCIS that characteristically lacks E-cadherin [43,44]. MMPs are postulated to be important in the degradation of basement membrane and extracellular matrix during cancer cell invasion. In particular, MMP9 and MMP2 (also know as gelatinases) are involved in the degradation of type 4 collagen that comprises basement membranes, a process thought to be important in the development of invasive carcinoma and metastasis. Although degradation of basement membranes is not a function that is concordant with the usual clinical behaviour of LCIS (a non-invasive, low-risk cancer precursor), we suspect that the activity of MMP9 in LCIS must be tightly regulated. Indeed, MMP2 and MMP9 are secreted as zymogens and cleaved to their active form, and their activity is tightly regulated by several mechanisms, including the actions of tissue inhibitors of metalloproteinases (TIMPS). It is certainly conceivable that the expression of activated MMP9 helps effect the characteristic, permeative, 'Indian file' growth pattern of invasive lobular carcinoma. Our study indicates that MMP9 RNA and protein is already present at the precursor (LCIS) stage of invasive lobular carcinoma, and raises the possibility that factors leading to its activation may play a role in the development of invasive disease. MMP9 therefore represents an interesting therapeutic [32] and chemopreventive target for patients with lobular neoplasia, both invasive and non-invasive.

Finally, we note that the down regulation of cell junction proteins (such as E-cadherin and claudin 4) and up regulation of MMP9 that we observed may be taken by some to be evidence of so-called epithelial-mesenchymal transition in LCIS. Indeed, the transcription factor *Twist *is known to down regulate E-cadherin expression in invasive lobular carcinoma, and is postulated to be a master regulator of epithelial-mesenchymal transition and metastasis [45,46]. Although we agree that the abnormalities we have elucidated in LCIS in this study do overlap with those described *in vitro *in epithelial-mesenchymal transition, LCIS remains a cytokeratin-positive, epithelial neoplasm.

## Conclusions

In conclusion, an unbiased analysis of the LCIS transcriptome by SAGE has identified MMP9 overexpression and claudin 4 underexpression. This LSAGE library is publicly available at the Cancer Genome Anatomy Project SAGE website [20], and should facilitate further, much needed research on this enigmatic lesion.

## Abbreviations

ALH: atypical lobular hyperplasia; bp: base pair; CGAP: cancer genome anatomy project; CGH: Comparative Genomic Hybridisation; DCIS: ductal carcinoma in situ; DGED: Digital Gene Expression Displayer; H & E: haematoxylin & eosin; IDC: invasive ductal carcinoma; LCIS: lobular carcinoma in situ; LSAGE: long serial analysis of gene expression; MMP: matrix metalloproteinase; OCT: optimal controlled temperature medium; RT-PCR: reverse transcriptase polymerase chain reaction; SAGE: serial analysis of gene expression; TIMPS: tissue inhibitors of metalloproteinases.

## Competing interests

The authors declare that they have no competing interests.

## Authors' contributions

PA, DC and SS conceived of the study. PA drafted the manuscript. KP carried out the SAGE data analysis. MKH, CID, JH, HN and ADM carried out the immunohistochemical analysis. XW and SS carried out the quantitative RT-PCR. NK carried out macrodissection of LCIS and benign tissue used for quantitative RT-PCR. All authors read and approved the final manuscript.

## Supplementary Material

Additional file 1 A TIF file containing a figure of the macrodissection of LCIS from frozen tissue of the index case. (a) On low power of frozen section guide slide, the LCIS areas are easily distinguished from the benign epithelium. (b) The area circled, which is enriched for LCIS, was cut out of the OCT-embedded frozen tissue block. RNA was extracted from this tissue for SAGE. Click here for file

Additional file 2 A TIF file containing a figure of the Digital Northern blot of *E-cadherin *expression in breast L-SAGE libraries. Note that the LCIS library shows lower expression of *E-cadherin *than all of the other normal breast epithelial libraries. Click here for file

Additional file 3 A TIF file containing a figure of the Digital Northern blot of MMP2 expression in breast L-SAGE libraries. Note that the LCIS library shows higher MMP2 expression than the other normal breast luminal epithelial libraries, but less expression than the myoepithelial cell library and several stromal libraries. Click here for file
