# Supplementary figures and images for: Serial analysis of gene expression of lobular carcinoma in situ identifies down regulation of claudin 4 and overexpression of matrix metalloproteinase 9
Source: Breast Cancer Res. 2008 Oct 27;10(5):R91. doi: 10.1186/bcr2189 (PMC2614499; doi:10.1186/bcr2189)

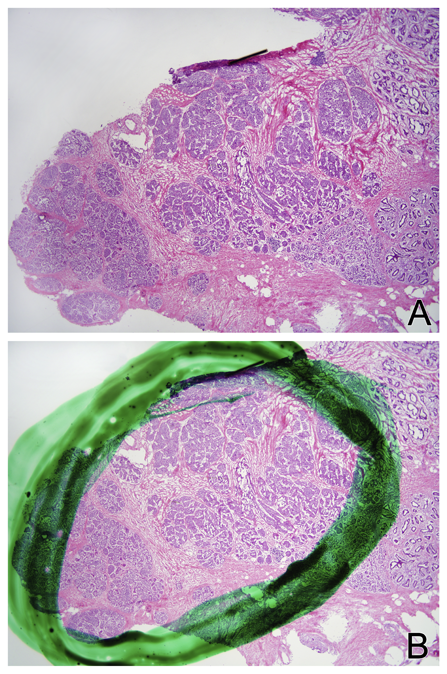

Supplement: Additional file 1 — A TIF file containing a figure of the macrodissection of LCIS from frozen tissue of the index case. (a) On low power of frozen section guide slide, the LCIS areas are easily distinguished from the benign epithelium. (b) The area circled, which is enriched for LCIS, was cut out of the OCT-embedded frozen tissue block. RNA was extracted from this tissue for SAGE. [file bcr2189-S1.tiff]

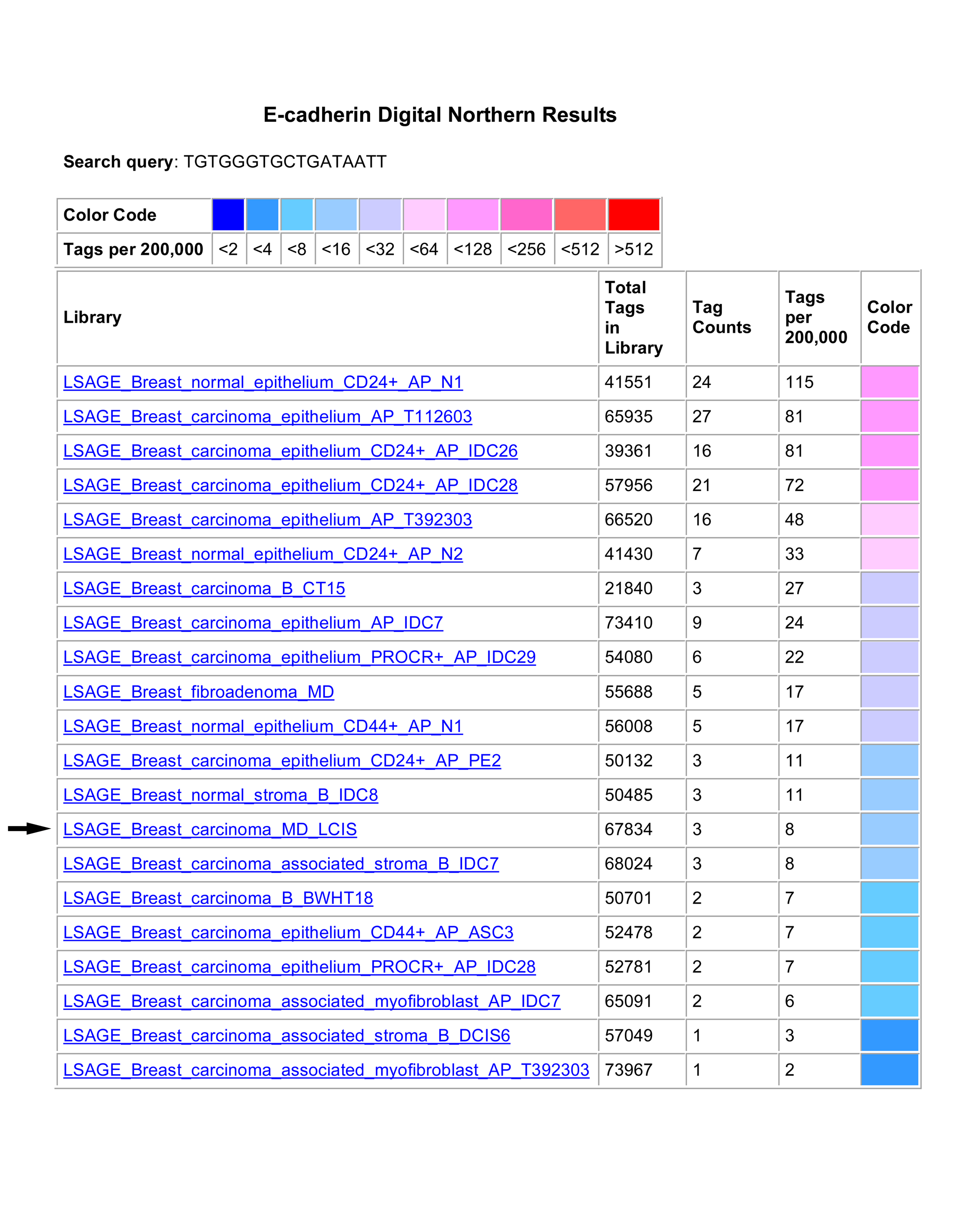

Supplement: Additional file 2 — A TIF file containing a figure of the Digital Northern blot of E-cadherin expression in breast L-SAGE libraries. Note that the LCIS library shows lower expression of E-cadherin than all of the other normal breast epithelial libraries. [file bcr2189-S2.tiff]

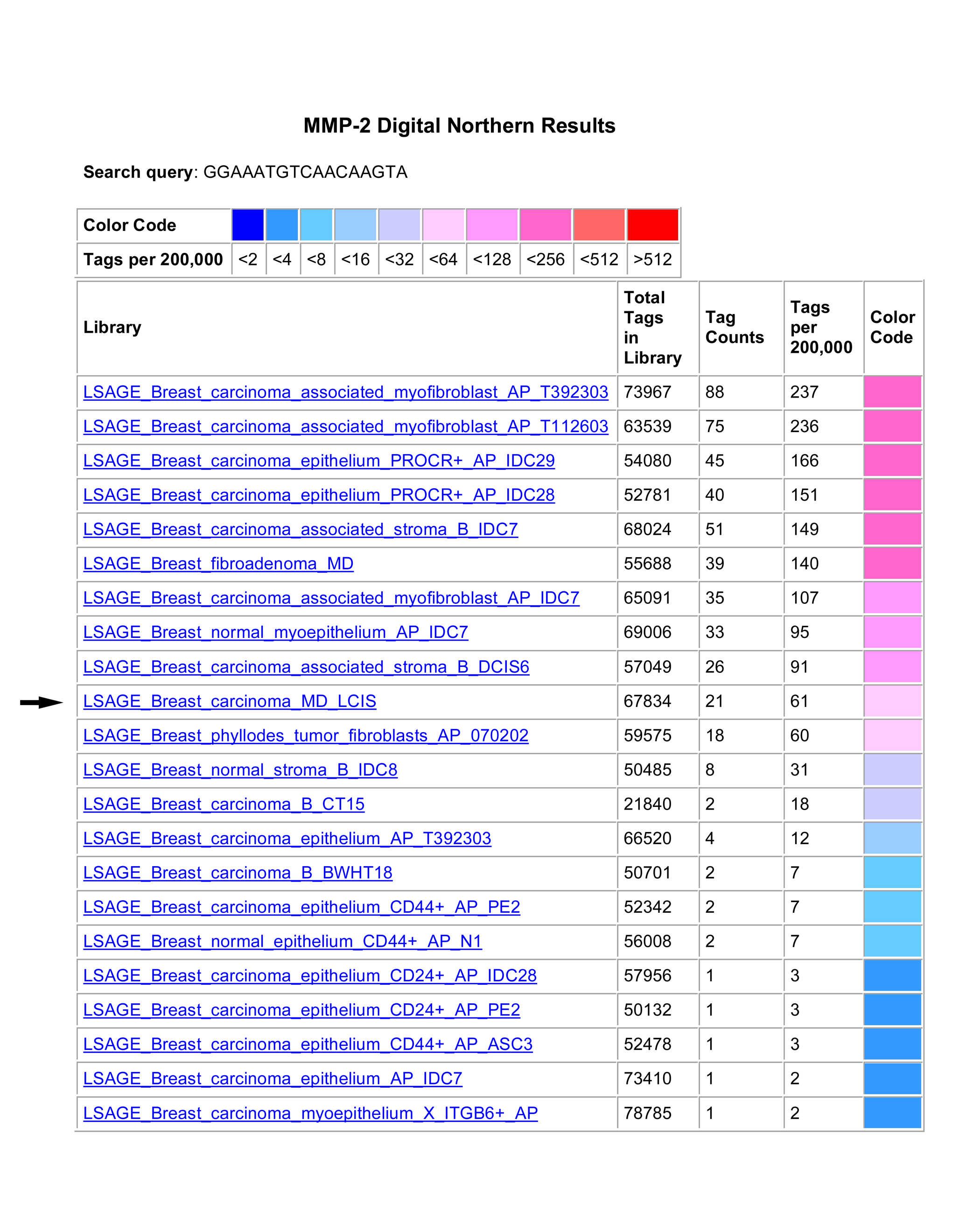

Supplement: Additional file 3 — A TIF file containing a figure of the Digital Northern blot of MMP2 expression in breast L-SAGE libraries. Note that the LCIS library shows higher MMP2 expression than the other normal breast luminal epithelial libraries, but less expression than the myoepithelial cell library and several stromal libraries. [file bcr2189-S3.tiff]
